# Ultrastructural and Immunohistochemical Alterations in Muscle and Vascular Tissues in Patients with Omphalocele

**DOI:** 10.3390/ijms27031460

**Published:** 2026-02-01

**Authors:** Dina Rosca-Al Namat, Adrian Romulus Rosca, Delia Hînganu, Ludmila Lozneanu, Fabian Cezar Lupu, Elena Hanganu, Elena Tarca, Jana Bernic, Nadia Al Namat, Razan Al Namat, Marius Valeriu Hînganu

**Affiliations:** 1 Faculty of Medicine, Grigore T. Popa University of Medicine and Pharmacy, University Street No. 16, 700115 Iasi, Romania; dina.rosca-al.namat@umfiasi.ro (D.R.-A.N.); romulus-adrian.v.rosca@umfiasi.ro (A.R.R.); ludmila.lozneanu@umfiasi.ro (L.L.); hanganu.elena1@umfiasi.ro (E.H.); tarca.elena@umfiasi.ro (E.T.); marius.hinganu@umfiasi.ro (M.V.H.); 2 Department of Mechanical, Mechatronics and Robotics Engineering, Mechanical Engineering Faculty, Gheorghe Asachi Technical University of Iasi, 700050 Iasi, Romania; fabian-cezar.lupu@academic.tuiasi.ro; 3 Discipline of Pediatric Surgery, “Nicolae Testemitanu” State University of Medicine and Pharmacy, MD-2004 Chisinau, Moldova; jana.bernic@usmf.md; 4 Faculty of Medicine, Dunarea de Jos University of Galati, 800008 Galati, Romania; nadia.alnamat@gmail.com; 5 Faculty of Dental Medicine, Apollonia University, 700511 Iasi, Romania; dr.razan_romania@yahoo.com

**Keywords:** omphalocele, omphalocele molecular mechanism, scanning electron microscopy, congenital abnormalities, developmental myology

## Abstract

Omphalocele is a congenital abdominal wall defect whose underlying muscular and fascial structural characteristics remain incompletely understood. This study aimed to investigate the anatomical and ultrastructural features of the abdominal wall in patients with omphalocele to provide additional insight into tissue organization at the defect margins. We report a series of 11 term-born patients diagnosed with omphalocele between 2024 and 2025 who were admitted to a pediatric surgery department for operative management. Following informed consent from legal guardians, two small intraoperative biopsies were obtained during surgical repair from the rectus abdominis muscle and its anterior aponeurosis, sampled from the supraumbilical and subumbilical regions. Tissue specimens were fixed within 48 h and analyzed using conventional histopathology and scanning electron microscopy to assess potential structural and ultrastructural alterations. The combined microscopic approaches allowed for a detailed evaluation of muscle and aponeurotic architecture in different abdominal regions. These observations contribute to a more comprehensive understanding of abdominal wall tissue characteristics in omphalocele and may support improved interpretation of the structural changes associated with this congenital condition.

## 1. Introduction

Omphalocele is a congenital malformation of the anterior abdominal wall characterized by herniation of abdominal viscera through the umbilical ring, covered by a membranous sac composed of amnion, Wharton’s jelly, and peritoneum. With an estimated incidence of approximately 1 per 5000–7000 live births, omphalocele remains a significant cause of neonatal morbidity and mortality, particularly because more than 70% of affected infants present with associated structural or chromosomal anomalies [1,2,3]. Despite advances in prenatal diagnosis and neonatal surgical management, the biological mechanisms underlying the formation of this defect remain incompletely understood.

Classical embryological theories have attributed omphalocele to either failure of the physiological return of the midgut into the abdominal cavity following transient herniation between the 6th and 10th gestational weeks or to abnormal lateral folding of the embryonic body wall resulting in incomplete fusion of mesodermal layers [4,5]. While these models account for the gross anatomical presentation of the defect, they do not fully explain its regional predilection, frequent association with other malformations, or the heterogeneity observed in abdominal wall tissue organization.

More recent developmental concepts have expanded these classical models by incorporating vascular and molecular mechanisms. Emerging evidence suggests that aberrant vasculogenesis, transient ischemic events, or impaired mesodermal proliferation may critically influence early abdominal wall development, particularly in the supraumbilical region [6]. These mechanisms emphasize the role of regional perfusion and tissue oxygenation during early embryogenesis, shifting the focus from purely morphogenetic failure to dynamic disturbances affecting tissue maturation.

During early development, particularly between the 4th and 6th gestational weeks, formation of the anterior abdominal wall coincides with extensive remodeling of the embryonic vascular network. Physiological midgut herniation and its subsequent reintegration into the abdominal cavity are accompanied by coordinated changes in arterial supply and venous drainage [7,8]. Disruption of this finely regulated process may result in spatially restricted hypoperfusion, with downstream effects on myogenic differentiation and extracellular matrix organization. Within this context, a regionally confined alteration of arterial configuration—conceptualized as an “arterial switch phenomenon”—has been proposed as a unifying mechanism linking vascular instability, local tissue hypoxia, and impaired structural maturation of the abdominal wall [9].

The anterior abdominal wall is a complex anatomical structure whose integrity depends on coordinated development of muscle fibers, connective tissue layers, and their supporting vascular network. Normal myogenesis requires adequate oxygen delivery and timely vascular maturation, while extracellular matrix composition and collagen organization are essential for tensile strength and mechanical stability. Perturbations affecting any of these components during critical developmental windows may predispose to structural weakness and failure of wall closure.

In this setting, direct analysis of abdominal wall tissues from patients with omphalocele offers a unique opportunity to explore the biological substrates of the defect. However, existing studies have primarily focused on macroscopic anatomy or clinical outcomes, with relatively limited attention to the microscopic, molecular, and ultrastructural characteristics of the affected tissues. In particular, the relationship between vascular architecture, muscle fiber maturation, and extracellular matrix composition at the margin of the defect remains insufficiently characterized [10].

To address this gap, we conducted a combined histological, immunohistochemical, and ultrastructural investigation of intraoperatively collected tissue samples from patients with omphalocele. A targeted panel of molecular markers was selected to assess three complementary biological axes relevant to abdominal wall development: vascular density and maturation (*CD31*, *CD34*, *ICAM-2*), angiogenic signaling and hypoxic response (*VEGFR*), and muscular and extracellular matrix development (*DUX4*, Collagen I). This integrated approach enables a detailed examination of the vascular–muscular–stromal interface at the edge of the abdominal wall defect, a region predicted by embryological models to exhibit the earliest pathological alterations.

The aim of the present study was to characterize the muscle and fascial components of the anterior abdominal wall in patients with omphalocele using combined immunohistochemical and ultrastructural methods. By analyzing rectus abdominis muscle and anterior rectus sheath biopsies obtained from the margin of the defect, we sought to determine whether the observed structural features are more consistent with a primarily vascular-driven mechanism of abdominal wall formation failure or with a primary defect of mesodermal or muscular differentiation. Through this analysis, we aim to refine current embryological concepts of omphalocele pathogenesis by integrating evidence from vascular biology, developmental myology, and extracellular matrix organization.

## 2. Results

### 2.1. Scoring Results from Theimmunohistochemistry Study

The immunohistochemical scores for the analyzed markers are summarized in Table 1 (supraumbilical samples) and Table 2 (subumbilical samples), highlighting regional differences in vascular and stromal expression evaluated semi-quantitatively (0–3+).

#### 2.1.1. *ICAM-2* Immunohistochemical Scores

**Figure 1:**
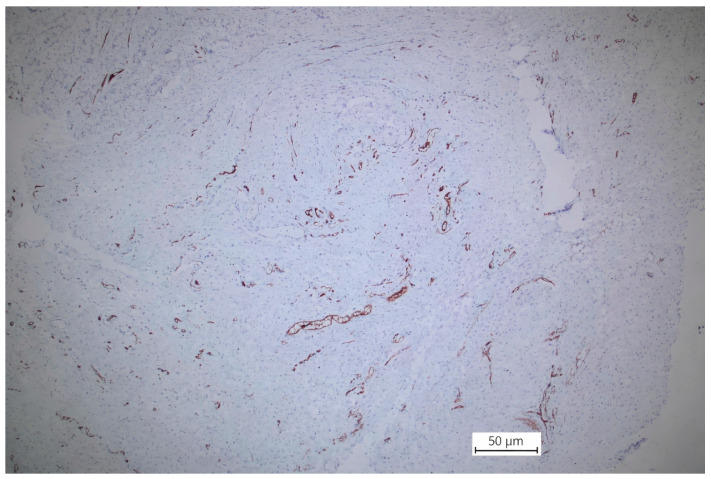
Supraumbilical region: ICAM—intermuscular vascular endothelium, membranous staining (anti-ICAM, 40×. Scale bar = 50 µm).

**Figure 2:**
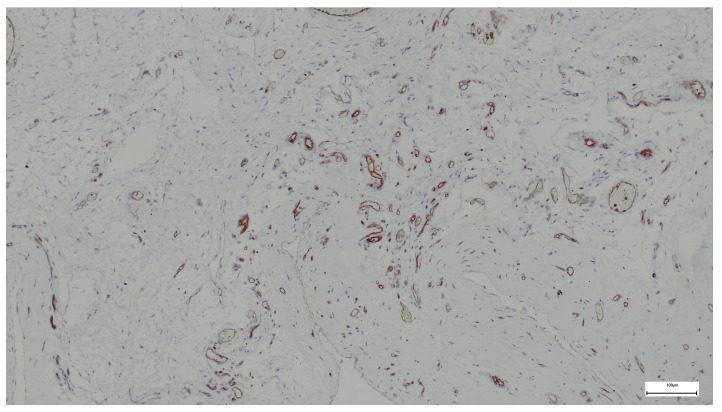
Subumbilical region: ICAM—intermuscular vascular endothelium, membranous staining (anti-ICAM, 40×. Scale bar = 100 µm).

Expressed as median and interquartile range (IQR), were identical in the supraumbilical and subumbilical regions, with median values of 2 in both areas. Comparison of paired samples using the Wilcoxon signed-rank test (non-parametric) showed no statistically significant difference between regions (U = 18.0, *p* = 0.361). These findings indicate the absence of regional differences in *ICAM-2* expression (Figure 1 and Figure 2).

#### 2.1.2. *CD31* Immunohistochemical Scores

**Figure 3:**
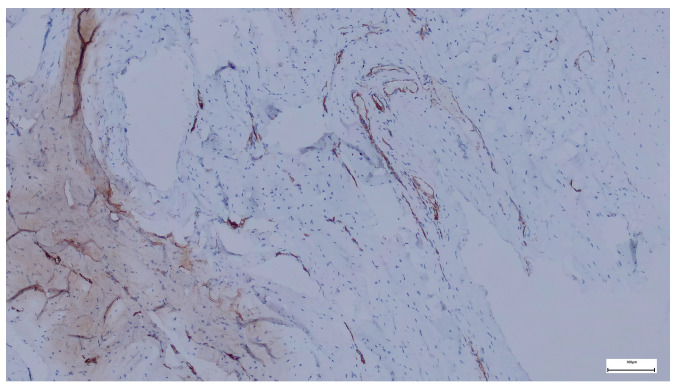
Supraumbilical region: *CD31*—vascular endothelial cells (*anti-CD31* 40×. Scale bar = 100 µm).

**Figure 4:**
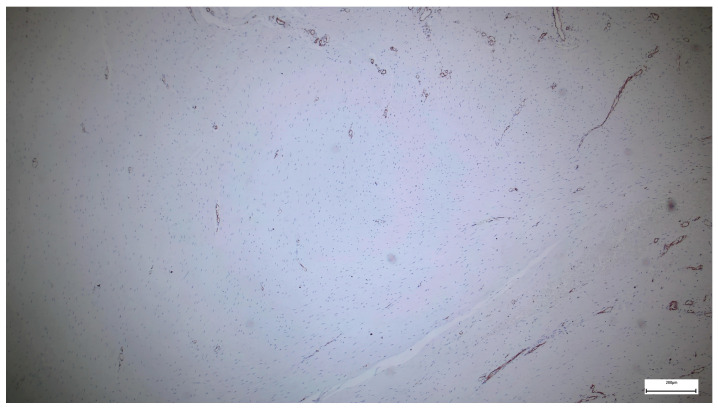
Subumbilical region: *CD31*—vascular endothelial cells (*anti-CD31* 40×. Scale bar = 200 µm).

Reported as median and IQR, were 2 in the supraumbilical region and 2 in the subumbilical region. Paired comparison using the Wilcoxon signed-rank test (non-parametric) revealed no statistically significant difference between regions (U = 13.0, *p* = 0.762), indicating the absence of significant regional variation in *CD31* expression (Figure 3 and Figure 4).

#### 2.1.3. *CD34* Immunohistochemical Scores

**Figure 5:**
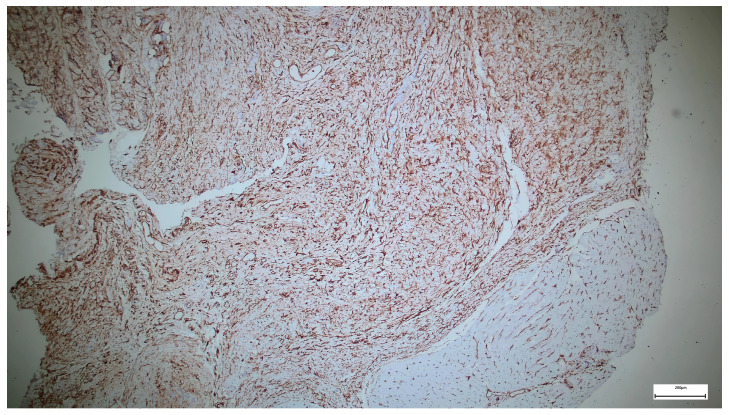
Supraumbilical region: *CD34*—vascular endothelial cells. Membranous staining (anti-*CD34* 40×. Scale bar = 200 µm).

**Figure 6:**
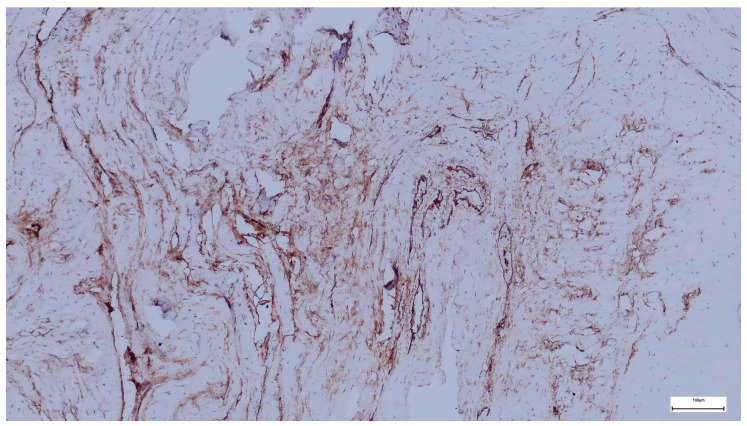
Subumbilical region: *CD34*—vascular endothelial cells. Membranous staining (*anti-CD34* 40×. Scale bar = 100 µm).

Expressed as median and IQR, were 2 in the supraumbilical region and 3 in the subumbilical region. Paired comparison using the Wilcoxon signed-rank test (non-parametric) showed a strong trend toward higher *CD34* expression in the subumbilical region, reaching the limit of statistical significance (U = 5.0, *p* = 0.0518). Effect size analysis using Cliff’s delta indicated a large magnitude of difference (δ ≈ −0.67), favoring the subumbilical region. Together, these findings suggest a biologically meaningful increase in subumbilical *CD34* expression, despite marginal statistical significance (Figure 5 and Figure 6).

#### 2.1.4. Collagen I Immunohistochemical Scores

Immunohistochemical staining for Collagen I in the supraumbilical region demonstrates strong and widespread extracellular positivity, predominantly distributed within the fascial and stromal compartments. The staining highlights thick, densely packed collagen bundles arranged in irregular, often concentric or whorled patterns around vascular and interstitial structures.

Collagen I immunoreactivity is particularly intense in the perivascular and interstitial connective tissue, outlining vessel walls and extending into the surrounding stroma. The collagen bundles appear coarse and heterogeneously organized, with variable orientation and limited evidence of uniform lamellar alignment. In several areas, collagen deposition is prominent despite a relatively loose cellular background.

**Figure 7:**
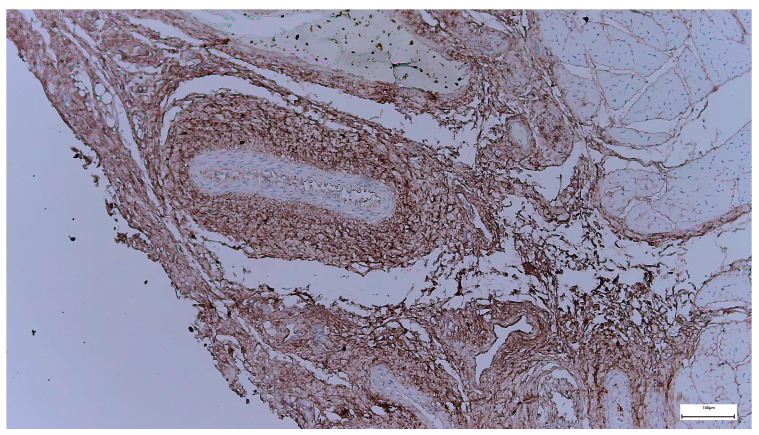
Collagen I immunohistochemistry in the supraumbilical region showing strong extracellular staining within fascial and perivascular connective tissue, with irregularly arranged and thick collagen bundles consistent with dysregulated matrix remodeling (40×. Scale bar = 100 µm).

Overall, the staining pattern indicates a high abundance of Collagen I, with marked regional variability in fiber thickness and spatial organization (Figure 7).

Immunohistochemical staining for Collagen I in the subumbilical region reveals diffuse and intense extracellular positivity, involving large areas of the connective tissue matrix. Collagen I highlights a dense but finely distributed fibrillar network, extending throughout the stroma and surrounding numerous vascular structures of variable caliber.

**Figure 8:**
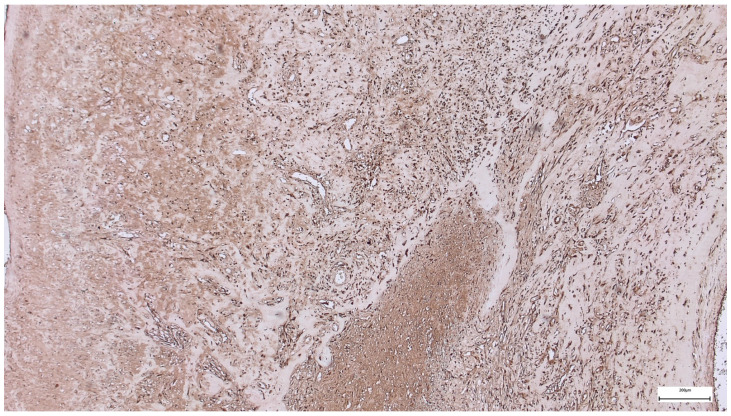
Collagen I immunohistochemistry in the subumbilical region showing diffuse extracellular staining with a finely reticular collagen network surrounding vascular and stromal structures, consistent with ongoing matrix remodeling rather than compact fibrotic organization (40×. Scale bar = 200 µm).

In contrast to the supraumbilical region, collagen bundles in the subumbilical samples appear thinner and more evenly dispersed, forming a complex, interlacing network rather than thick, compact lamellae. The staining outlines both perivascular connective tissue and interstitial stromal compartments, with collagen fibers frequently following tortuous or branching trajectories (Figure 8).

Overall, Collagen I expression is abundant and widespread, yet the matrix displays a more reticular and less compact architecture, with intervening spaces between fiber bundles and limited evidence of dense lamellar stacking.

Collagen I immunohistochemical scores, expressed as median and IQR, were high in both regions, with median values of 3 in the supraumbilical region and 2.5 in the subumbilical region. Paired comparison using the Wilcoxon signed-rank test (non-parametric) revealed no statistically significant difference between regions (U = 19.5, *p* = 0.383). These results indicate similarly elevated Collagen I expression in both supraumbilical and subumbilical areas, without evidence of regional variation.

#### 2.1.5. *DUX4* Immunohistochemical Scores

Immunohistochemical staining for *DUX4* in the supraumbilical region reveals predominantly nuclear immunoreactivity within cells of the connective tissue and adjacent muscular compartments. *DUX4*-positive nuclei are scattered and variably distributed, with a generally moderate staining intensity, forming a heterogeneous pattern across the examined field.

**Figure 9:**
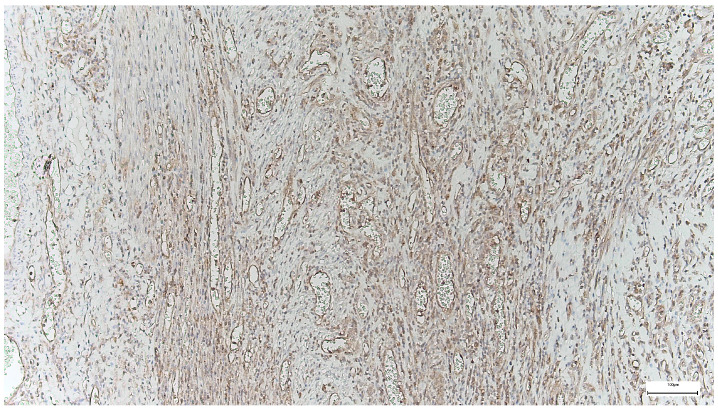
DUX4 immunohistochemistry in the supraumbilical region showing heterogeneous nuclear positivity in stromal and perivascular cells, consistent with delayed or incomplete tissue maturation rather than primary myopathic change (40×. Scale bar = 100 µm).

The immunoreactivity is not confined to a single cellular population, but is observed in elongated spindle-shaped cells within the stromal matrix, as well as in cells located in close spatial relationship to vascular structures. The overall distribution does not suggest diffuse, uniform expression, but rather a patchy nuclear positivity, interspersed among *DUX4*-negative cells. No overt architectural distortion or features of overt muscle fiber degeneration are evident in association with *DUX4*-positive areas (Figure 9).

In the subumbilical region, immunohistochemical staining for *DUX4* demonstrates more extensive nuclear positivity compared with the supraumbilical samples. *DUX4*-positive nuclei are observed in a larger proportion of stromal and mesenchymal cells, with moderate to strong staining intensity distributed across broad areas of the tissue section.

The nuclear labeling is particularly evident within spindle-shaped cells of the connective tissue, as well as in cells located at the interface between fascial structures and adjacent adipose tissue. The staining pattern remains predominantly nuclear, without diffuse cytoplasmic accumulation, and shows a relatively homogeneous distribution within the subumbilical field.

**Figure 10:**
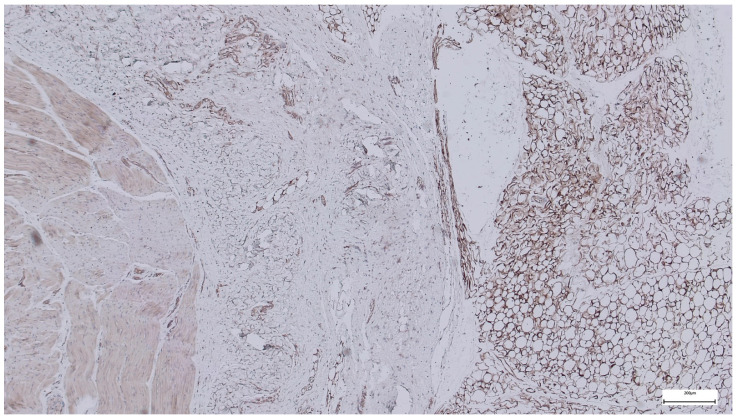
*DUX4* immunohistochemistry in the subumbilical region showing widespread nuclear positivity in stromal and mesenchymal cells, consistent with persistent transcriptional immaturity and delayed tissue maturation (40×. Scale bar = 200 µm).

Despite the increased extent of *DUX4* positivity, the surrounding tissue architecture does not display features of overt muscle fiber degeneration or dystrophic change (Figure 10).

*DUX4* immunohistochemical scores, expressed as median and interquartile range (IQR), were 2 in the supraumbilical region and 3 in the subumbilical region. Paired comparison using the Wilcoxon signed-rank test (non-parametric) did not demonstrate a statistically significant difference between regions (U = 8.5, *p* = 0.190). However, effect size estimation using Cliff’s delta indicated a moderate difference favoring higher subumbilical expression (δ ≈ −0.43). These findings suggest a trend toward increased *DUX4* expression in the subumbilical region, although this did not reach statistical significance.

Comparison of IHC scores between the supraumbilical (*n* = 5) and subumbilical (*n* = 6) regions using the Wilcoxon signed-rank test (paired, non-parametric) test showed similar values for *ICAM-2* (median 2 in both groups; *p* = 0.361) and *CD31* (median 2 in both groups; *p* = 0.762), indicating no regionally relevant differences for these markers. In contrast, *CD34* displayed higher sub-umbilical scores (median 3) compared with supraumbilical (median 2), with a strong trend toward significance (*p* = 0.052) and a large effect size, suggesting increased microvascular density/activation in the sub-umbilical segment. Collagen I remained globally elevated in both regions (supramedian 3 vs. submedian 2.5), without significant differences (*p* = 0.383), and *DUX4* showed a tendency toward higher subumbilical expression (median 3 vs. 2), though not statistically significant in this sample (*p* = 0.190). Overall, the results support a predominantly vascular regional difference (*CD34*) in the subumbilical area, within the context of relatively homogeneous expression for the inflammatory/endothelial markers examined.

### 2.2. Results of the SEM Study

Scanning electron microscopy of the supraumbilical region in omphalocele reveals a markedly altered extracellular matrix architecture. The tissue surface is composed of thick, irregular collagen lamellae arranged predominantly in a longitudinal direction, with evident lamellar delamination and fragmentation. Instead of a compact, well-organized fibrillar pattern, the collagen appears flattened, disrupted, and heterogeneously distributed, with abrupt transitions between denser and more porous areas.

**Figure 11:**
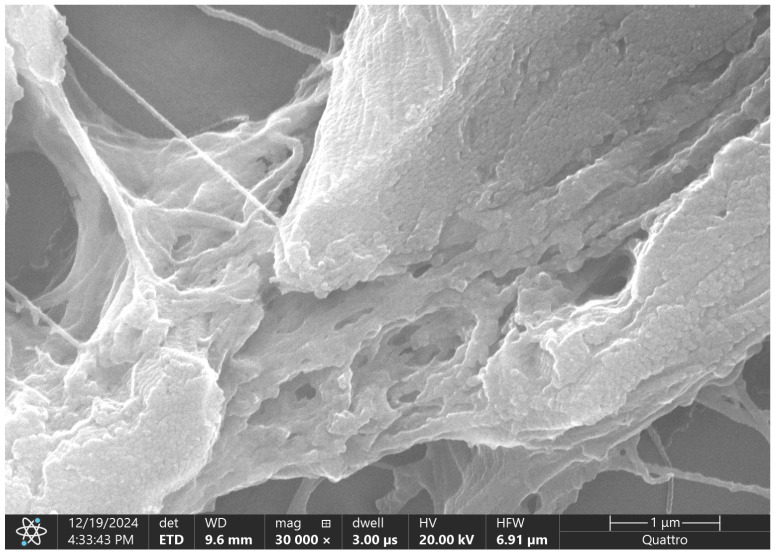
Scanning electron microscopy of the supraumbilical region in omphalocele showing disrupted extracellular matrix architecture with delaminated and fragmented collagen lamellae, irregular fibrillar organization, and microdiscontinuities consistent with incomplete matrix maturation (×30,000).

Multiple microdiscontinuities and interlamellar clefts are visible, indicating reduced cohesion between adjacent collagen layers. Fine, tensioned fibrillar strands bridging larger collagen fragments are occasionally observed, suggesting the presence of residual or incompletely assembled fibrils within an overall immature matrix. At this magnification, the architecture lacks the uniformity and dense packing characteristic of a mature fascial extracellular matrix (Figure 11).

Scanning electron microscopy of the subumbilical region demonstrates an extracellular matrix architecture that is structurally more open and filamentous compared with the supraumbilical samples. The matrix is composed of elongated, thin fibrillar elements forming a loose, interconnected network rather than compact lamellar sheets. Collagen fibers appear slender, variably oriented, and frequently bridged by delicate filamentous connections, creating a reticular pattern.

**Figure 12:**
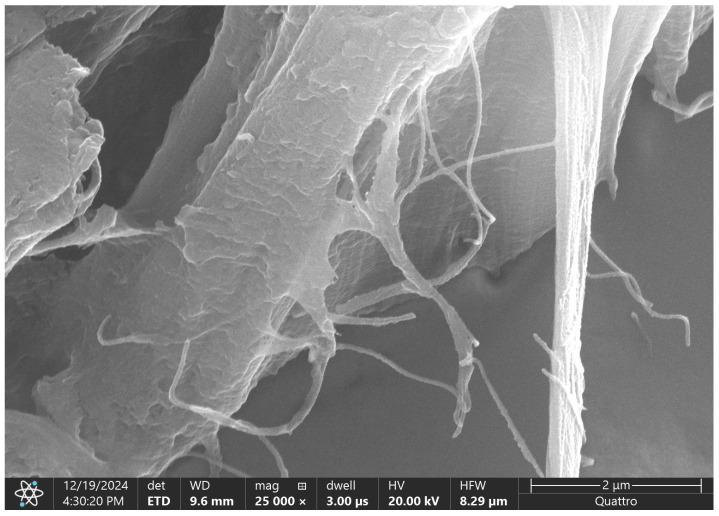
Scanning electron microscopy of the subumbilical region in omphalocele showing a loosely organized extracellular matrix composed of thin, elongated collagen fibers with reticular interconnections, consistent with an immature but actively remodeling matrix architecture (×25,000).

In several areas, the collagen bundles display reduced thickness and limited lateral fusion, with intervening spaces and microcavities between adjacent fibers. The surface morphology is smoother and less compacted than in supraumbilical regions, and clear lamellar stacking is largely absent. Overall, the ultrastructural appearance suggests a matrix that is less condensed but more dynamically interconnected (Figure 12).

### 2.3. Scoring Results from the SEM Study

Composite SEM scoring categories were defined according to standard practice as follows: scores of 0–2 were classified as low, scores of 3 as mild, scores of 4 as moderate, and scores of 5–6 as high/severe (Table 3).

Descriptive statistics for SEM indicated a bimodal distribution, characterized by a plateau of mild cases (score 3) and a distinct group with moderate to severe involvement (scores 4–6) (Table 4).

In Table 5, the data demonstrate a clear increase (“jump”) in severity beginning after Case 6, distinguishing a lower-severity subgroup (Cases 1–6) from a higher-severity subgroup (Cases 7–11).

Cases 1–6 (all with a score of 3) exhibit a consistent morphological phenotype characterized by mild alterations. The absence of variability (SD = 0) suggests either highly similar tissue characteristics across cases or a stable, steady-state pathological process.

Cases 7–11 (scores 4–6) demonstrate progressive morphological worsening, with a peak severity observed in Case 8. The internal heterogeneity of the group (SD ≈ 0.84) suggests different stages of involvement or differences between supra- and subumbilical regions that are reflected in the overall score.

Compared with cases 1–6, cases 7–11 are significantly more morphologically affected (mean difference ≈ 1.8 points).

Correlation with the IHC score (0–3) can be seen in Table 6 and Table 7.

In the supraumbilical region:

- Pro-endothelial/angiogenic profile demonstrates they *CD31* and *CD34* exhibited moderate expression on average (≈2), but with notable variability (SD = 0.71), largely driven by differences between Case 5 (score 1) and Case 1 (score 3).
- Fibrosis/extracellular matrix (ECM): Collagen I expression was high (mean 2.8, median 3) and relatively homogeneous across cases.
- Inflammation/Cell Adhesion: *ICAM-2* was moderately high, ranging between scores 2 and 3.
- *DUX4* Expression: *DUX4* levels were moderate (≈2.4), with no extreme values observed.

In the subumbilical region:

- Inflammation/Cell Adhesion (*ICAM-2*): *ICAM-2* expression was uniform across all cases (score = 2), indicating consistent levels of inflammation/adhesion in the subumbilical region.
- Pro-endothelial/Angiogenic Profile (*CD31*, *CD34*): *CD34* levels were high and homogeneous (≈2.8–3), reflecting a robust microvascular density. *CD31* was moderate on average (mean 2.17) but showed some variability (1–3), likely reflecting differences in endothelial maturity or vessel continuity.
- Fibrosis/Extracellular Matrix (Collagen I): Collagen I expression was moderately high (mean 2.5) with some variation (SD = 0.55), suggesting differences in fibrosis between cases.
- *DUX4* Expression: *DUX4* was generally high (median 3), with only a single lower value observed (Case 10 = 2), indicating consistent upregulation in most cases.

Comparison of mean marker values between the supra- and subumbilical regions revealed several notable patterns. *ICAM-2* expression was largely similar between regions, with only a slight decrease in the subumbilical area (Δ = −0.20), indicating comparable levels of inflammation/adhesion. *CD31* showed a modest subumbilical increase (Δ = +0.17), whereas *CD34* exhibited a more pronounced rise (Δ = +0.83), suggesting substantially greater microvascularization and neoangiogenic activity in the subumbilical region. DUX4 expression was also slightly higher subumbilically (Δ = +0.43), indicating potentially stronger activity or expression in this area. In contrast, Collagen I was somewhat higher in the supraumbilical region (Δ = −0.30), reflecting a tendency toward more pronounced fibrosis and extracellular matrix remodeling above the umbilicus.

### 2.4. SEM and IHC Correlation

In this cohort, SEM scores were uniform for Cases 1–6 (all = 3) and variable for Cases 7–11 (range 4–6). The lack of variation in the supraumbilical region (Cases 1–5) precluded any meaningful correlation with IHC markers, as statistical association requires variability. Similarly, *ICAM-2* was constant in the subumbilical region (all = 2), preventing correlation with SEM-B. Therefore, reliable correlation could only be explored for markers that exhibited variability within the subumbilical subset.

Observed trends in the subumbilical region were primarily descriptive. *CD31* did not increase in cases with higher SEM scores; for example, Case 9 exhibited high SEM (5) but low *CD31* (1), suggesting that SEM may reflect structural network degradation rather than endothelial density. *CD34* values were near saturation (many cases scored 3) across the variable SEM range, producing essentially no observable correlation due to a ceiling effect. Collagen I appeared in both scores 2 and 3 at high SEM, indicating fibrosis presence but without a stable linear relationship to SEM severity. *DUX4* expression was similarly near saturation, with only Case 10 showing a lower value (2 at SEM 4), implying that *DUX4* may indicate cellular presence or activation rather than the degree of structural alteration.

Overall, quantitative correlations between SEM and IHC markers were limited. Uniform SEM in the supraumbilical region and saturated markers subumbilically (*ICAM-2*, *CD34*, *DUX4*) resulted in weak or undefined associations.

Integrating SEM and IHC observations, cases with increasing SEM scores (7–11) exhibited consistently high subumbilical microvascularization (*CD34*), potentially reflecting compensatory neoangiogenesis. Endothelial signal (*CD31*) was variable, likely influenced by structural degradation observed in SEM. Fibrosis was moderately high in both regions, slightly more pronounced supraumbilically (Collagen I). *DUX4* expression was higher subumbilically but did not correlate proportionally with SEM, indicating that it may reflect cellular activity rather than structural severity.

## 3. Discussion

This study proposes the idea that an arterial “switch” caused by the anatomical herniation of the primitive intestine and its return into the abdomen around the 6th week of gestation may play a role in the pathogenesis of the defect. At that point, the original vascular branches may be replaced by others, leading to insufficient vascular development and impaired fusion of the abdominal wall. We will be able to support or refute this hypothesis based on our results.

The theory starts from the idea that, around the 6th week, physiological herniation and subsequent reintegration of the intestinal loops alter the local arterial network: some vascular trunks regress or are replaced, a chaotic reorganization of the circulation occurs, and a region of the abdominal wall becomes hypovascular/hypoxic, preventing proper fusion and maturation of the wall.

The integrative etiopathogenic model already formulated acknowledges early vasculogenesis and hypoxia (the 4–6-week window) as a common core mechanism underlying abdominal wall defects.

The selected IHC panel translates this vascular hypothesis into measurable morphological parameters along three axes:

- Vascular axis: *VEGFR*, *CD31*, *CD34*, *ICAM-2*
- Muscular axis: *DUX4* (muscle dysgenesis/maturation)
- Extracellular matrix axis: Pro-Collagen III α1 and Collagen I (immature vs. mature ECM)

Furthermore, the fact that the samples are collected in a standardized manner from the margin of the defect—supra- and subumbilical, from the sheath and from the muscle—allows us to determine whether the vascular/muscular/fibrous profile is segmental and related to a specific “perfusion map”, which is essential for supporting the possibility of a regional arterial switch.

*VEGFR*, *CD31*, *CD34*, and *ICAM-2* were chosen specifically to separate three dimensions of vascular pathology:

- How many vessels there are,
- How mature they are,
- How strongly they respond to hypoxia/reactive angiogenesis.

What would support our arterial switch hypothesis (regional ischemia at 6 weeks): *CD31*/*CD34*:

- Altered microvascular density (reduced or disorganized) in the critical zones at the edge of the defect, compared with control regions or with other areas of the same abdominal wall;
- And possible discrepancies between supra- and subumbilical regions that may suggest different perfusion territories.

Increased *VEGFR* in the same territories indicates reactive angiogenesis in response to hypoxia—meaning tissue that has been “left behind” by the normal vascular network and is attempting to compensate belatedly through neovascularization.

Low/patchy *ICAM-2*, while *CD31* is present, indicates numerous but immature, unstable vessels—typical of a network that has been “rearranged” and insufficiently matured, rather than a territory that simply never developed vessels (as in a very early embryologic defect).

A high *CD31+*/*ICAM-2*—ratio at the margin of the defect fits a scenario of pathological angiogenesis secondary to an arterial switch rather than a simple “abdominal cavity too small” problem or a strict defect of lateral fold fusion.

What would contradict the arterial switch hypothesis is globally low vascular density, without increased *VEGFR* and with few *CD31+*/*CD34+* vessels throughout the wall. This would support a primary mesodermal defect (theories 2 and 5) rather than an arterial redistribution event and a similar vascular profile in areas with and without the defect, with no supra/sub or sheath/muscle gradient, which would argue against a regional mechanism linked to vascular branching.

*DUX4* was included specifically to determine whether the rectus abdominis muscle is intrinsically dysgenetic or merely a “victim” of defective perfusion.

If *DUX4* is strongly and diffusely expressed, including in areas with relatively preserved vascularization, then there is evidence for primary muscular dysgenesis (Theory 5—a defect of muscular differentiation), which cannot be explained solely by hypoxia or an arterial switch.

If *DUX4* expression is high only in territories with a pathological vascular profile (*VEGFR*↑, *CD31*/*CD34* disorganized, *ICAM-2*↓), then we can argue that dysgenesis and delayed fiber maturation are secondary to regional hypoxia—thus supporting a vascular theory (arterial switch during herniation/re-entry).

In this way, *DUX4* becomes the marker that tells you whether the muscle is “born abnormal” or “damaged by circulation”.

The ECM markers were chosen to assess whether the wall defect is primarily fibro-mesodermal or the result of incomplete maturation due to ischemia.

Pro-Collagen III α1 ↑ and Collagen I ↓, together with immature matrix with predominance of type III collagen, suggest:

- Either a primary defect of synthesis/remodeling (the connective dysgenesis theory),
- Or an “emergency remodeling” response in hypoxic tissue that fails to progress to mature type I collagen.

If the regions with altered vascular profiles are also those with a low Col I/Col III ratio, we can argue that abnormal vascularity precedes and determines the defective maturation of the sheath—again supporting a mechanism of arterial switch/regional hypoxia.

Conversely, if this collagen imbalance is diffuse and does not correlate with vascular abnormalities, the vascular hypothesis weakens, and the “collagen theory” (primary ECM defect) gains weight.

The IHC panel allows you to differentiate between these mechanisms as follows:

1. Evidence in favor of an arterial switch/vascular mechanism during physiological herniation (rotation/blockage theory + vascular component):
  - Regional gradient of vascular density (*CD31*/*CD34*),
  - *VEGFR*↑ and *ICAM-2*↓ in critical territories,
  - *DUX4* and Col I/Col III ratio altered in the same territories, not diffusely.
2. Evidence in favor of an early embryologic/mesodermal defect (Theory 2 + early molecular model):
  - Diffuse muscular hypoplasia and immature sheath,
  - Reduced vascular density but without significant signs of reactive angiogenesis (modest *VEGFR*, globally low *ICAM-2*, no gradient).
3. Evidence in favor of primary muscular/connective dysgenesis (Theory 5):
  - Diffuse *DUX4*↑ regardless of vascular status,
  - Col I/Col III imbalance present even in tissues with near-normal vascularization.

The strong Collagen I expression observed in the supraumbilical region reflects a tissue environment characterized by advanced extracellular matrix deposition but incomplete structural maturation. Although Collagen I is typically associated with mature and mechanically robust connective tissue, the irregular and disorganized arrangement of collagen bundles seen here suggests that matrix accumulation has occurred in a non-physiological or dysregulated manner.

When interpreted in conjunction with the SEM findings—showing delaminated and fragmented collagen lamellae—the Collagen I immunoprofile supports the notion that matrix quantity does not equate to matrix quality. Rather than forming a compact, uniformly oriented scaffold, collagen appears excessively deposited yet poorly integrated into a coherent architectural framework.

Within the context of the proposed vascular-driven developmental model, this pattern may represent a secondary or compensatory fibrotic response to early tissue stress, such as transient hypoperfusion or hypoxia during a critical window of abdominal wall development. In such conditions, fibroblastic activity and collagen synthesis may be initiated or amplified, while the processes responsible for proper fiber alignment, cross-linking, and mechanical integration remain impaired.

Importantly, the coexistence of high Collagen I expression with ultrastructural features of matrix instability argues against a simple primary collagen deficiency. Instead, it supports a scenario in which vascular disturbance precedes and shapes extracellular matrix remodeling, leading to the formation of a structurally weak but collagen-rich abdominal wall in the supraumbilical region.

In the supraumbilical region, Collagen I is abundantly expressed but exhibits a disorganized spatial arrangement, indicating excessive yet mechanically inefficient matrix deposition.

The Collagen I expression pattern observed in the subumbilical region suggests a matrix that is rich in mature collagen but remains structurally dynamic and incompletely consolidated. Although Collagen I is present in high amounts, its relatively fine, reticular organization indicates ongoing remodeling rather than the formation of a rigid, mechanically integrated scaffold.

When compared with the supraumbilical region—where Collagen I forms thick, irregular, and compact bundles—the subumbilical matrix appears less fibrotic and more adaptable, consistent with a tissue environment that continues to undergo reorganization. This architectural difference aligns with the ultrastructural SEM findings, which demonstrate a more filamentous and interconnected extracellular matrix umbilically.

Within the framework of the proposed vascular-driven developmental model, this pattern may reflect relatively preserved or compensatory vascularization in the subumbilical region, allowing sustained collagen synthesis and remodeling while delaying full structural maturation. In such a context, collagen deposition proceeds, but the transition toward a fully aligned and mechanically optimized matrix remains incomplete.

Importantly, the coexistence of abundant Collagen I with a loose and heterogeneous organization supports the concept that matrix composition and matrix architecture are dissociated in omphalocele. These findings argue against a primary deficiency of collagen production and instead favor a mechanism in which vascular and perfusion-related factors influence the tempo and quality of extracellular matrix maturation.

Compared with the supraumbilical region, the subumbilical area exhibits abundant but more finely distributed Collagen I, forming a reticular matrix architecture indicative of continued remodeling rather than dense fibrotic consolidation.

The presence of nuclear *DUX4* immunoreactivity in the supraumbilical region is interpreted as a marker of delayed or incomplete myogenic and mesenchymal maturation, rather than as an indicator of primary muscle pathology. In contrast to its well-established pathogenic role in adult-onset muscular dystrophies, where *DUX4* is aberrantly reactivated in fully differentiated myofibers, the expression observed here occurs within a developmental and perinatal tissue context.

The heterogeneous and regionally restricted pattern of *DUX4* positivity suggests that its expression is not constitutive or diffuse, but instead reflects localized areas in which cells have retained an immature transcriptional profile. This interpretation is consistent with experimental and developmental studies demonstrating that *DUX4* activity is physiologically compatible with early myogenic or mesodermal states and is normally silenced during later stages of muscle differentiation.

Within the framework of the proposed vascular-driven model, the supraumbilical *DUX4* expression may reflect a secondary response to impaired local perfusion or tissue hypoxia, conditions known to delay myogenic maturation and to maintain cells in a less differentiated state. Importantly, the absence of widespread or intense *DUX4* expression, together with the lack of morphological features suggestive of dystrophic muscle damage, argues against a primary myopathic process.

Taken together, the supraumbilical *DUX4* staining pattern supports the concept that the muscle and stromal tissues at the margin of the defect are developmentally delayed rather than intrinsically dysgenetic, reinforcing the hypothesis that vascular instability during a critical embryological window contributes to the observed structural abnormalities.

In the supraumbilical region, *DUX4* expression is heterogeneous and nuclear, suggesting persistence of an immature transcriptional state rather than diffuse pathological reactivation.

The broader and more uniform *DUX4* expression observed in the subumbilical region suggests a persistent immature transcriptional state affecting a larger tissue compartment than in the supraumbilical region. Rather than indicating primary muscle pathology, this pattern is more consistent with delayed or dysregulated tissue maturation within a developmental context.

In contrast to the patchy, regionally restricted *DUX4* positivity seen supraumbilically, the subumbilical distribution appears more diffuse, implying that a wider area of tissue has remained in a less differentiated state. This finding is compatible with the concept that subumbilical tissues may experience prolonged or altered developmental signaling, potentially influenced by local vascular dynamics.

When interpreted alongside the ultrastructural findings—characterized by a loosely organized, filamentous extracellular matrix—and the immunohistochemical evidence of increased microvascular density in the subumbilical region, the *DUX4* expression profile supports a model of ongoing but incomplete maturation rather than fixed developmental arrest. In this scenario, improved or compensatory vascularization may permit continued matrix remodeling and cellular survival, while simultaneously maintaining cells in a transcriptionally immature state.

Importantly, the absence of diffuse, intense *DUX4* expression within mature myofibers, together with the lack of histological features of muscular dystrophy, argues against a primary myopathic mechanism. Instead, the subumbilical *DUX4* pattern reinforces the hypothesis that vascular and oxygenation-related factors modulate the tempo of myogenic and mesenchymal maturation, contributing to regional heterogeneity within the abdominal wall.

Compared with the supraumbilical region, the subumbilical area shows a broader and more homogeneous *DUX4* expression pattern, suggesting region-dependent differences in the regulation of tissue maturation.

The justification for this panel is that it transforms the “arterial switch” hypothesis from a merely plausible embryologic explanation into a testable morpho-molecular hypothesis, by finely correlating vessel type and maturity (*VEGFR*, *CD31*, *CD34*, *ICAM-2*), muscle status (*DUX4*), and fibro-sheath maturity (Pro-Collagen III, Collagen I) in samples taken precisely from the region where such a switch would have been expected to occur.

Importantly, the interpretation of *DUX4* expression in the present study differs fundamentally from its established pathogenic role in adult-onset muscular dystrophies. In FSHD, *DUX4* reactivation occurs in fully differentiated myofibers and triggers aberrant transcriptional programs leading to muscle degeneration. By contrast, the tissue analyzed here derives from a developmental context, in which transient *DUX4* activity is physiologically compatible with early myogenic or mesodermal states. Therefore, the observed *DUX4* immunoreactivity is interpreted not as a marker of muscle damage, but as an indicator of incomplete myogenic maturation or persistence of an immature transcriptional profile [11,12,13,14].

Within the framework of fetal myogenesis, progression from undifferentiated mesenchymal progenitors toward mature myofibers is tightly regulated by spatial and temporal cues, including vascular supply and oxygen tension. Experimental data indicate that hypoxia and altered perfusion can delay myogenic differentiation and maintain cells in a less mature transcriptional state. In this regard, the regional persistence of *DUX4* expression, particularly when associated with ultrastructural features of immature muscle and extracellular matrix remodeling, may reflect a developmental delay linked to local vascular constraints rather than a fixed myopathic process [15,16,17,18,19,20,21].

Nevertheless, *DUX4* was used in this study as an exploratory and non-canonical marker of developmental immaturity, and not as a definitive indicator of myogenic stage. The absence of parallel assessment of established myogenic differentiation markers (such as *MyoD*, *myogenin*, *desmin*, or embryonic/fetal myosin heavy chain isoforms) represents a limitation. Future studies integrating *DUX4* with canonical myogenic markers and quantitative transcriptomic approaches will be required to more precisely define its role within the spectrum of fetal muscle maturation in omphalocele [22,23,24].

The ultrastructural features observed in the supraumbilical region are consistent with incomplete maturation of the extracellular matrix rather than established, stable fibrosis. Lamellar separation, surface fragmentation, and irregular collagen organization indicate that matrix deposition has occurred, but that subsequent processes of fibril alignment, compaction, and structural consolidation have remained impaired.

Such a pattern is compatible with a developmental environment characterized by transient hypoperfusion or hypoxia, in which extracellular matrix synthesis is initiated but fails to progress toward full structural maturity. In this context, the persistence of disorganized collagen lamellae may reflect an arrest at an intermediate stage of connective tissue development, rather than a primary absence of matrix formation.

Within the framework of the proposed arterial switch hypothesis, occurring during the period of physiological midgut herniation and return (approximately the sixth gestational week), these ultrastructural alterations may represent the morphological imprint of a critical window of vascular instability. A regional reduction or reorganization of blood supply at this stage could disrupt oxygen and nutrient delivery, thereby impairing coordinated extracellular matrix remodeling in the developing abdominal wall.

Importantly, the lack of a dense, mechanically integrated collagen network suggests that the abdominal wall in omphalocele is not simply incomplete in quantity, but structurally weakened in quality. This supports the concept that vascular disturbances during early development may precede and determine the formation of a mechanically insufficient wall, rather than representing a secondary phenomenon. The SEM findings therefore complement the immunohistochemical evidence of altered vascular and matrix-related markers and provide ultrastructural support for a vascular-driven mechanism contributing to omphalocele pathogenesis.

The subumbilical ultrastructural pattern is indicative of an immature but actively remodeling extracellular matrix, characterized by thin, loosely arranged collagen fibers rather than dense, delaminated lamellae. This architecture is compatible with a tissue environment in which matrix deposition is ongoing, but consolidation into a mechanically robust structure has not yet been achieved.

In contrast to the supraumbilical region—where collagen appears flattened, fragmented, and lamellarly separated—the subumbilical matrix displays features suggestive of active fibrillogenesis and remodeling, including fine inter-fibrillar connections and a more reticular organization. These differences support the presence of regional heterogeneity in matrix maturation, potentially reflecting distinct local developmental or perfusion conditions.

Within the framework of the arterial switch hypothesis, the subumbilical region may have been exposed to relative preservation or secondary recovery of vascular supply, allowing continued matrix synthesis and reorganization despite overall developmental delay. The presence of thin, elongated collagen fibers is consistent with an environment influenced by angiogenic activity and ongoing tissue adaptation, rather than fixed structural failure.

Taken together, the subumbilical SEM features suggest that extracellular matrix abnormalities in omphalocele are not uniform across the abdominal wall, but instead follow a regional pattern consistent with differential vascular influences. This observation complements the immunohistochemical findings of increased microvascular density in the subumbilical region and supports the concept that vascular dynamics play a central role in shaping the ultrastructural phenotype of the abdominal wall.

### 3.1. Benchmark Analysis in Relation to Previously Reported Data

Benchmarking the present findings against previously published reference data allows a more precise positioning of our results within the current framework of tissue remodeling and vascular pathology research. Recent histopathological and immunohistochemical studies investigating congenital or developmentally altered tissues have consistently reported increased microvascular density accompanied by stromal disorganization and incomplete extracellular matrix maturation [25,26,27,28].

In agreement with these benchmarks, our results demonstrate an extensive vascular network highlighted by *CD31* and *CD34* immunostaining, supporting the presence of an active angiogenic microenvironment. However, when compared with benchmark studies conducted in postnatal or adult tissues—where vascular structures tend to display a more uniform caliber and organized spatial distribution [29]—the samples analyzed in the present study exhibit marked vascular heterogeneity and irregular endothelial arrangement. These differences are most likely attributable to the developmental stage of the tissue and to adaptive remodeling mechanisms specific to rare congenital pathological contexts [30].

The use of semi-quantitative immunohistochemical scoring represents a limitation of the present study and may introduce a degree of subjectivity. Although standardized criteria and intra-patient comparisons were applied, digital image-based quantification would provide greater objectivity and sensitivity for detecting subtle differences. Future studies integrating automated image analysis are required to validate and extend the present findings.

With regard to the stromal compartment, the expression pattern of Collagen I further differentiates our findings from established benchmarks of mature fibrotic remodeling. Rather than a dense and compact collagen architecture, characteristic of chronic fibrosis, the analyzed tissues display a loose and disorganized collagen distribution, suggesting an ongoing and dynamically regulated remodeling process. This pattern reflects stromal plasticity rather than terminal fibrotic transformation and underscores the developmental specificity of the observed changes.

Although this adapted SEM technique provides reliable information on extracellular matrix architecture and surface morphology, it does not allow detailed intracellular ultrastructural analysis.

Overall, benchmarking our results against established histological and immunohistochemical reference models reveals both convergent features and distinctive characteristics. These differences do not indicate methodological discrepancies but instead highlight the unique biological context of the analyzed pathology. Consequently, the present findings extend existing benchmark data by providing insight into the interplay between angiogenic activation and stromal remodeling in a rare congenital setting. The limited cohort size restricts statistical power and increases the risk of type II errors, particularly for correlation analyses between immunohistochemical and ultrastructural parameters. Therefore, the absence of statistically significant correlations should be interpreted with caution and does not exclude biologically relevant associations.

Because omphalocele is a rare condition, one of the main limitations of our study was the small number of patients. To address this, we initiated a multicenter collaboration with additional hospitals, which allowed us to obtain a larger patient cohort and to apply the same study materials and methodology across all centers. The small size of intraoperative biopsies represents an inherent limitation and may not fully capture local tissue heterogeneity. Although sampling was standardized and performed at anatomically equivalent, non-scarred regions, selection was based on intraoperative visual assessment. Therefore, a degree of sampling bias cannot be excluded and should be considered when interpreting the results.

The robustness of the statistical inference is limited by the small cohort size and precludes application of advanced multivariate or machine learning–based analytical approaches. Integration of SEM and immunohistochemical data was therefore performed using exploratory, non-parametric methods appropriate for ordinal variables. Future studies with larger datasets and quantitative metrics will be required to enable more complex analytical modeling.

This study has several limitations that require caution in mechanistic interpretation. First, the cohort is small (*n* = 11) and single-center, and the absence of a comparable control group (including the potential differences introduced by the use of post-mortem samples) limits the ability to attribute the observed vascular and matrix alterations specifically to omphalocele. Second, some of the markers proposed to test the vascular hypothesis (e.g., *VEGFR*, *ICAM-2*, *Pro-Collagen IIIα1*) are not yet reported consistently in the quantitative results, and the semi-quantitative 0–3 scoring shows ceiling/saturation effects for certain markers, limiting robust correlations with ultrastructural parameters. In addition, the topographic differences between supra- and subumbilical regions should be interpreted as trends rather than definitive evidence until a paired per-patient design and objective quantification (digital microvessel density, percentage of positive area, ECM indices) are implemented.

Given these constraints, the present study should be regarded as exploratory and hypothesis-generating, providing a structural and biological framework for future quantitative validation in larger, multicenter cohorts.

The absence of an external control group represents a limitation of this study; however, the paired intra-patient design partially compensates for this constraint and is ethically appropriate in the context of neonatal congenital anomalies.

Complete blinding to anatomical location was not feasible due to inherent morphological differences between sampled regions, and residual observer bias cannot be excluded. In addition, potential confounding variables such as gestational age and associated anomalies were not included in multivariable analyses due to the limited cohort size.

The cross-sectional design of the present study provides a static view of tissue architecture and does not capture dynamic developmental or reparative processes. Integration of molecular profiling or functional assays in future studies will be essential to further elucidate the mechanisms underlying the observed alterations.

In the future, these limitations will be addressed through multicenter expansion of the cohort, inclusion of appropriate control groups, and standardized quantitative analyses (with blinded assessment and inter-observer reproducibility), as well as by integrating more canonical markers of muscle maturation and direct indicators of the hypoxic response. Within this framework, the hypothesis of an “arterial switch” may be considered a plausible explanation compatible with some of the observed patterns, but it cannot yet be asserted as a demonstrated causal mechanism.

### 3.2. Future Directions and Quantitative Refinement

Although the present study provides a detailed semi-quantitative and ultrastructural characterization of vascular, muscular, and extracellular matrix alterations at the margin of omphalocele defects, future investigations will benefit from the integration of objective digital image analysis to further refine these observations. Specifically, the assessment of microvascular density expressed as number of vessels per mm^2^, together with quantitative measurements of positively stained area (%) and digitally derived H-scores, would allow a more precise evaluation of regional vascular and stromal differences.

Such analyses should be performed using standardized regions of interest (ROIs), selected at predefined anatomical locations (supra- and subumbilical margins) and analyzed at fixed magnifications to ensure reproducibility across cases and centers. Reporting the number of analyzed fields per section, the total number of quantified vessels, and the criteria used for vessel identification would further enhance methodological transparency.

The application of dedicated digital pathology software platforms (e.g., ImageJ-based workflows or commercially available whole-slide image analysis systems) would also facilitate automated or semi-automated quantification, reduce observer-dependent variability, and enable more robust correlations between immunohistochemical markers and ultrastructural parameters. In the context of rare congenital anomalies such as omphalocele, where patient numbers are inherently limited, such quantitative standardization is particularly important to maximize the analytical value of small cohorts and to allow meaningful comparisons across future multicenter studies.

## 4. Materials and Methods

This observational, single-center study included patients with omphalocele who underwent surgical repair, during which tissue samples were collected for histology, immunohistochemistry (IHC), and scanning electron microscopy (SEM). We analyzed a series of 11 patients diagnosed with omphalocele between 2024 and 2025 who were admitted to the Pediatric Surgery Department for operative management. All intraoperative tissue sampling was performed by the same surgical team, consisting of experienced pediatric surgeons trained in neonatal abdominal wall reconstruction. Biopsies were obtained using a standardized protocol, with identical anatomical landmarks and sampling depth in all cases, in order to minimize inter-operator variability.

The study was approved by the institutional ethics committee of Grigore T Popa University of Medicine and Pharmacy Iasi, Romania, number 373 from 2 January 2024. And complied with the principles of the Declaration of Helsinki (2013). All tissue was anonymized. Informed consent was secured from each patient’s legal representative.

During surgery, two small biopsies—each measuring only a few millimeters—were obtained from each patient. The samples were collected from the rectus abdominis muscles and their anterior aponeuroses, with one biopsy taken from the supraumbilical margin and the other from the infraumbilical margin of the defect. Patients were eligible if biopsies from both regions could be safely obtained. Demographic, perinatal, and surgical data were recorded.

All specimens were submitted to the pathology (anatomical pathology) department for evaluation of potential structural alterations, where the biopsy samples were fixed within 48 h. In addition to standard histopathological examination, the samples underwent scanning electron microscopy (SEM) analysis to provide a detailed ultrastructural assessment.

### 4.1. Sample Collection and Tissue Procurement

Tissue samples were coded prior to immunohistochemical and ultrastructural evaluation, and scoring was performed according to predefined criteria, without access to individual clinical data.

#### 4.1.1. Tissue Sampling Protocol in Patients Operated for Omphalocele

Tissue sampling was performed in the same cohort of patients who underwent surgical repair for omphalocele and were included in the histological and immunohistochemical studies. Sampling followed a standardized protocol: full-thickness fascial and muscle fragments were obtained at supra- and subumbilical margins, immediately orient-ed, labeled, and fixed in formaldehyde.

Due to ethical constraints, no external healthy control tissue was included. Instead, a paired intra-patient design was employed, using supraumbilical and subumbilical samples from the same individuals as internal controls, allowing regional comparison under identical biological conditions.

#### 4.1.2. Surgical Approach and Incision

All procedures were carried out under general anesthesia, using a standard midline approach adapted to the size and position of the omphalocele sac. After disinfection and sterile draping of the operative field, a median supra- and subumbilical skin incision was performed, centered on the omphalocele defect. The incision circumscribed the base of the sac and the umbilical stump, allowing access to the herniated viscera and the underlying anterior abdominal wall.

The subcutaneous tissue was carefully divided in line with the skin incision, preserving vascular supply as much as possible. The omphalocele sac was then opened and excised as required for the planned repair. Once the edges of the parietal defect and the rectus abdominis muscles were clearly exposed, attention was directed to tissue sampling.

#### 4.1.3. Tissue Sampling Sites and Technique

**Figure 13:**
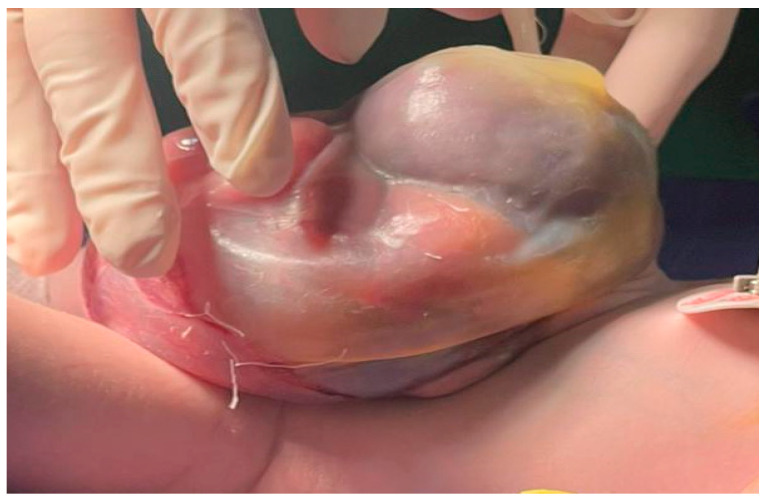
Clinical aspect of a giant omphalocele, showing the presence of the liver within the omphalocele sac.

**Figure 14:**
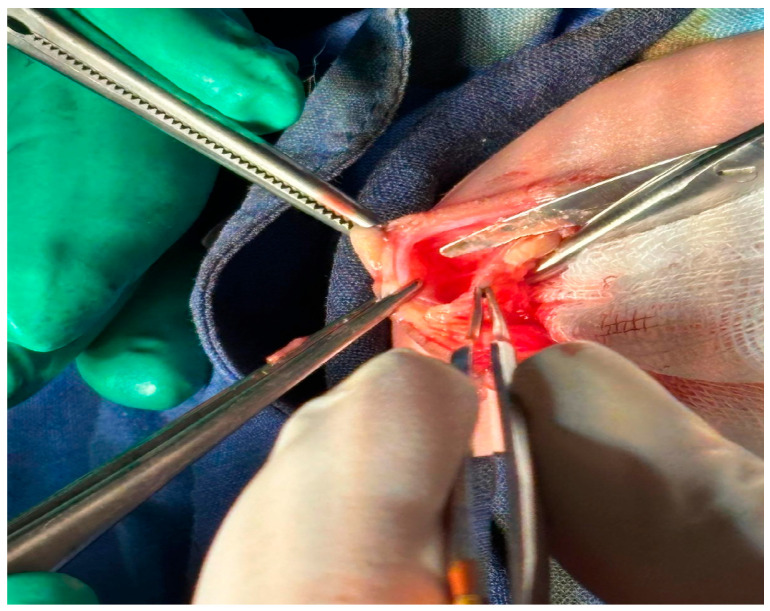
Intraoperative aspect showing the technique used for obtaining the muscle and aponeurosis biopsy.

Tissue specimens were obtained from both the anterior rectus sheath and the rectus abdominis muscle itself, at the margin of the abdominal wall defect, in order to capture the transition zone between the malformed and more normally developed parietal structures (Figure 13 and Figure 14).

Two levels were systematically sampled:

- Supraumbilical margin of the defect
- Subumbilical margin of the defect

At each level, a small, full-thickness fragment of the anterior rectus sheath was excised, together with an adjacent fragment of the underlying rectus abdominis muscle. The biopsies were taken from the immediate edge of the defect, avoiding areas of obvious scar tissue, necrosis, or previous sutures, so as to minimize artifacts and to ensure that the specimens reflected the native structural abnormalities of the abdominal wall.

The dimensions of the tissue fragments were chosen so as to provide sufficient material for histological, immunohistochemical, and ultrastructural studies, while avoiding any clinically relevant weakening of the abdominal wall. After sampling, meticulous hemostasis was obtained and the reconstructive steps of the abdominal wall repair were carried out according to the standard surgical plan in each case.

#### 4.1.4. Handling and Fixation of Specimens

Immediately after excision, all tissue samples were gently oriented, labeled according to patient ID and sampling site (supraumbilical vs. subumbilical; sheath vs. muscle), and transferred to the pathology containers. Specimens were fixed within 48 h in formaldehyde-based fixative and subsequently processed and embedded in paraffin according to routine histopathological procedures, in preparation for histology, immunohistochemistry, and additional ultrastructural assessment.

#### 4.1.5. Rationale for Sampling Strategy

The decision to sample the rectus sheath and rectus abdominis muscle at the edge of the parietal defect, both supra- and subumbilically, was based on the need to:

- Characterize the structural and maturation abnormalities of the abdominal wall musculature in omphalocele;
- Evaluate potential differences in composition and organization of the connective tissue and fascia in the transition zone between the defect and the surrounding abdominal wall;
- Obtain comparably located specimens across patients, ensuring reproducibility and allowing meaningful comparison of histological, immunohistochemical, and ultrastructural findings.

This standardized sampling protocol ensured that all specimens originated from anatomically equivalent regions directly involved in the malformation, while preserving the safety and integrity of the surgical repair.

No histological or clinical features suggestive of dystrophic muscle pathology were present.

### 4.2. Application of Specific Markers and Immunohistochemical Evaluation

IHC was performed on formalin-fixed, paraffin-embedded sections. Marker-specific protocols were applied:

1. *CD31* endothelial staining and semi-quantitative scoring.
2. *CD34* vascular/stromal staining.
3. *DUX4* myogenic marker staining.
4. Collagen I ECM staining.
5. *ICAM-2* endothelial integrity marker.

#### 4.2.1. Immunohistochemistry for *CD31*

Endothelial cell density and vascularization were evaluated using immunohistochemical staining for *CD31*. Formalin-fixed, paraffin-embedded tissue sections were processed following a standard preparation workflow, including deparaffinization and rehydration. Heat-induced epitope retrieval was performed in an acidic buffer (pH 6) according to the manufacturer’s guidelines.

For endothelial staining, a monoclonal antibody directed against CD31 (clone JC70A, Leica, Biosystems, Newcastle upon Tyne, UK) was applied at a 1:100 dilution. Sections were incubated with the primary antibody overnight, followed by the application of a polymer-based detection system, according to the recommended immunohistochemical procedure. Signal visualization was achieved through an enzyme-mediated chromogenic reaction, and nuclei were counterstained using a routine method. Slides were subsequently dehydrated and mounted permanently.

Appropriate positive and negative controls were included to confirm staining specificity and to ensure the validity of the immunohistochemical analysis.

CD31 staining was assessed in terms of:

- The extent of endothelial cell labeling,
- The intensity of immunoreactivity,
- The overall vascular density within the areas of interest.

A semi-quantitative scoring system may be applied, for example:

- 0—no detectable endothelial staining
- 1+—sparse, weakly positive vascular profiles
- 2+—moderate endothelial labeling
- 3+—abundant, strongly positive vascular structures

This assessment can be complemented by descriptive evaluation of vessel morphology or localization when relevant to the study objectives.

#### 4.2.2. Immunohistochemistry for *CD34*

The evaluation of vascular structures and stromal components was further complemented by immunohistochemical staining for *CD34*. Formalin-fixed, paraffin-embedded tissue sections were processed through a standard preparation sequence, including deparaffinization and rehydration. Heat-induced epitope retrieval was performed in an alkaline buffer (pH 9) following the manufacturer’s recommendations.

A monoclonal antibody directed against *CD34* (clone QBEnd-10, Dako, Glostrup, Denmark) was used at a 1:50 dilution. The sections were incubated with the primary antibody overnight, after which a polymer-based detection system was applied according to established immunohistochemical procedures. Visualization of the immunoreactive signal was achieved using a chromogenic enzymatic reaction, and nuclear counterstaining was performed using a routine method. The slides were then dehydrated and permanently mounted.

Appropriate positive and negative controls accompanied each staining run to confirm antibody specificity and exclude nonspecific reactions.

*CD34* staining was assessed by considering:

- The distribution and density of *CD34*-positive endothelial or stromal cells,
- The intensity of labeling (negative, weak, moderate, strong),
- The overall vascular profile within the examined tissue regions.

A semi-quantitative scoring system may be used, for example:

- 0—no detectable staining
- 1+—weak and sparsely distributed positive structures
- 2+—moderate staining with increased vascular density
- 3+—strong, widespread endothelial labeling

When relevant, morphological characteristics of vessels or stromal elements are described to support the interpretation of tissue architecture.

#### 4.2.3. Immunohistochemistry for *DUX4*

The assessment of *DUX4* expression was performed on formalin-fixed, paraffin-embedded tissue sections using immunohistochemistry. Sections underwent standard preparatory processing, including deparaffinization and rehydration, followed by heat-induced epitope retrieval in an alkaline buffer (pH 9), in accordance with the manufacturer’s recommendations.

For *DUX4* detection, a monoclonal antibody targeting *DUX4* (clone E5-5, Abcam Cambridge, UK) was applied at a 1:500 dilution. The sections were incubated with the primary antibody overnight, after which a polymer-based detection system was utilized according to the established immunohistochemical workflow. Visualization of the reaction product was achieved through an enzyme-mediated chromogenic method, and nuclei were counterstained using a routine laboratory procedure. Slides were subsequently dehydrated and permanently mounted.

Appropriate positive and negative controls were included to ensure the specificity and reliability of the staining.

*DUX4* immunoreactivity was assessed based on:

- The presence and distribution of nuclear *DUX4*-positive cells,
- The intensity of staining (negative, weak, moderate, strong),
- The overall pattern of expression within tissue compartments of interest.

A semi-quantitative scoring system may be employed, for example:

- 0—no detectable *DUX4* staining
- 1+—weak and focal nuclear positivity
- 2+—moderate nuclear labeling in a wider distribution
- 3+—strong, prominent nuclear expression

When relevant, the spatial relationship between *DUX4* positivity and specific tissue structures may also be described to support morphological interpretation.

*DUX4* was selected as an exploratory marker based on its documented role as a transcriptional regulator active during early human development, including preimplantation and early embryonic stages, where it participates in chromatin remodeling and activation of early developmental gene networks. While *DUX4* is best known for its pathological re-expression in adult skeletal muscle in facioscapulohumeral muscular dystrophy, accumulating evidence indicates that its physiological expression is temporally restricted to early developmental windows and is normally epigenetically silenced during later myogenic maturation. In this context, persistence or regional re-expression of *DUX4* in fetal or neonatal muscle tissue may reflect delayed or dysregulated myogenic differentiation rather than primary muscle pathology.

*DUX4* was not used as a staging marker of myogenic differentiation, but as an exploratory indicator of transcriptional immaturity.

#### 4.2.4. Immunohistochemistry for Collagen I

The evaluation of connective tissue architecture included immunohistochemical staining for Collagen I. Formalin-fixed, paraffin-embedded tissue sections were processed through a standard preparation protocol, comprising deparaffinization and rehydration, followed by heat-induced epitope retrieval in an alkaline buffer (pH 9), according to the manufacturer’s recommendations.

Detection of Collagen I was performed using a monoclonal antibody targeting the protein (clone EPR7785, Abcam Cambridge, UK), applied at a 1:1500 dilution. Sections were incubated with the primary antibody overnight, after which a polymer-based detection system was employed following established immunohistochemical procedures. Visualization of the reaction product was accomplished using an enzyme-mediated chromogenic method, and nuclear counterstaining was carried out routinely. Slides were subsequently dehydrated and permanently mounted.

Appropriate positive and negative controls were included with each staining run to ensure specificity and to validate the immunohistochemical results.

Assessment of Collagen I immunoreactivity included:

- The distribution of collagen labeling within stromal and connective tissue regions,
- The intensity of staining (negative, weak, moderate, strong),
- The overall pattern of extracellular matrix organization.

A semi-quantitative scoring approach may be applied, such as:

- 0—no detectable Collagen I staining
- 1+—weak, focal extracellular labeling
- 2+—moderate staining with broader deposition
- 3+—strong, widespread extracellular matrix positivity

Descriptive notes on collagen fiber density, orientation, or localization relative to tissue layers may be added to support interpretation.

#### 4.2.5. Immunohistochemistry for *ICAM-2*

The characterization of endothelial integrity and intercellular adhesion was further assessed using immunohistochemical staining for *ICAM-2.* Formalin-fixed, paraffin-embedded tissue sections were processed through a standard workflow that included deparaffinization and rehydration. Heat-induced epitope retrieval was performed in an acidic buffer (pH 6), following the manufacturer’s recommendations.

For ICAM-2 detection, a monoclonal antibody specific to *ICAM-2* (clone EPR19114-113, Abcam Cambridge, UK) was applied at a 1:4000 dilution. Sections were incubated with the primary antibody overnight, after which a polymer-based detection system was used in accordance with established immunohistochemical procedures. Chromogenic visualization was achieved through an enzyme-mediated reaction, and nuclei were counterstained by routine methodology. Slides were then dehydrated and permanently mounted.

Appropriate positive and negative controls were included in each staining series to verify specificity and ensure the reliability of the immunohistochemical reaction.

*ICAM-2* staining was assessed based on:

- The distribution of endothelial *ICAM-2* labeling,
- The intensity of staining (negative, weak, moderate, strong),
- The pattern of vascular or microvascular expression within regions of interest.

A semi-quantitative scoring system may be applied, such as:

- 0—no detectable *ICAM-2* staining
- 1+—weak, focal endothelial positivity
- 2+—moderate labeling with broader vascular representation
- 3+—strong, widespread endothelial expression

When relevant, descriptive notes regarding vessel morphology or spatial relationships may be included to support interpretation.

### 4.3. Ultrastructural Assessment of the Samples Using Scanning Electron Microscope (SEM)

**Figure 15:**
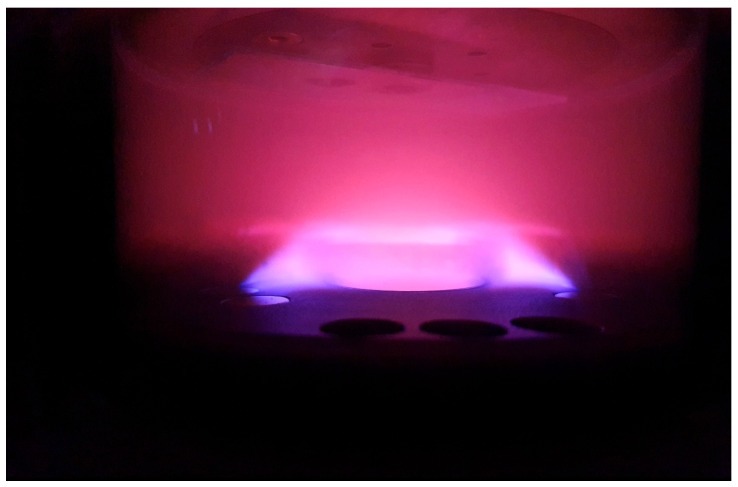
The image captures the moment in which the plasma field is formed during the application of the gold coating to the slide.

SEM)was performed on deparaffinized, dehydrated specimens coated with conductive film. Evaluation focused on collagen fiber organization, vascular impressions, and muscle fiber maturation (Figure 15).

The ultrastructural investigation was conducted on the same cohort of patients included in the histological and immunohistochemical studies. Tissue samples were obtained from the sheath and the rectus abdominis muscle of patients who underwent surgical repair of omphalocele, as well as from postmortem cases. All specimens were fixed in formaldehyde and subsequently embedded in paraffin.

Scanning electron microscopy is employed to analyze metallic, non-metallic, and biological materials, and can operate in three modes: high vacuum, low vacuum, and environmental SEM (ESEM). To obtain high-resolution images, the high-vacuum mode was selected. Samples were therefore prepared accordingly, including dehydration to ensure appropriate imaging conditions.

For scanning electron microscopy, tissue sections were obtained from conventionally stained hematoxylin–eosin (HE) slides after careful removal of the glass coverslip and exposure of the tissue surface. The specimens were re-fixed in 2.5% glutaraldehyde buffered with 0.1 M sodium cacodylate (pH 7.4), post-fixed in 1% osmium tetroxide, dehydrated through a graded ethanol series, and dried at the critical point using CO_2_. Samples were then mounted on aluminum stubs and sputter-coated with a 7 nm layer of gold prior to SEM analysis performed in high-vacuum mode using a secondary electron detector (VegaTescan LMH II, TESCAN, Brno, Czech Republic). This adapted SEM protocol applied to histological sections has been previously validated and described in detail [31].

A critical requirement for electron-based surface examination is that the specimen surface be electrically conductive; for this reason, the sections were coated with a thin, ap-proximately 7 nm layer of gold.

Analyses were performed using a state-of-the-art scanning electron microscope (SEM), the VegaTescan LMH II (TESCAN, Brno, Czech Republic). Technical features include: magnification capacity up to 100,000×, secondary electron (SE) detector, high-vacuum capability, tungsten filament, a carousel accommodating seven samples (standard size length × width × height in millimeters (10 × 10 × 45 mm) and the dedicated VegaTescan software package (version 4.2).

### 4.4. Statistical Analyses

A multimodal statistical framework was applied to determine convergence between IHC marker scoring and SEM ultrastructural characteristics.

#### 4.4.1. Descriptive Statistics

Ordinal IHC scores were summarized by median and interquartile range; SEM descriptors were categorized into ordered structural classes.

#### 4.4.2. Topographic Comparisons

Paired non-parametric tests examined systematic differences between supra- vs. subumbilical sites and between fascia vs. muscle.

Rank-based correlation methods (appropriate for ordinal paired datasets) evaluated associations between IHC indices and SEM-derived categories (e.g., collagen organization, vascular microarchitecture, muscle fiber maturity).

#### 4.4.3. Statistical Methods (IHC and SEM)

Immunohistochemical (IHC) scores were assessed semi-quantitatively on an ordinal scale from 0 to 3 (0 = negative, 1 = weakly positive, 2 = moderately positive, 3 = strongly positive), evaluated separately for the supraumbilical and subumbilical samples. For each marker, descriptive statistics (mean, standard deviation, and median) were calculated, and distributions between the two regions were compared using non-parametric tests appropriate for ordinal data. Differences between supra- and subumbilical regions were analyzed using a two-tailed Wilcoxon signed-rank test (paired, non-parametric) test, with statistical significance set at *p* < 0.05 and paired comparisons within the same patient.

For scanning electron microscopy, each case received a composite SEM score, calculated as the sum of several relevant ultrastructural parameters (e.g., organization and continuity of the collagen matrix, degree of fragmentation/porosity, and overall structural integrity), each rated on an ordinal scale. SEM scores were calculated separately for the supraumbilical and subumbilical regions and summarized using mean, standard deviation, and median values.

The association between the severity of ultrastructural alterations and the expression of IHC markers was assessed using Spearman’s correlation coefficient (ρ), appropriate for ordinal scoring and non-normal distributions. Results were interpreted based on the direction and magnitude of the correlation (positive/negative), and statistical significance was set at *p* < 0.05. Statistical analyses were performed using standard biostatistical software (e.g., SPSS - version 29.0, R - version 4.3.2, and GraphPad Prism - version 10.0), and data were reported as descriptive measures and non-parametric comparisons between groups.

Objective digital quantification (microvessel density/mm^2^, positive area percentage, H-score) was planned; however, due to the retrospective nature of the study and variability in available material, the present analysis relies on semi-quantitative scoring.

## 5. Conclusions

Taken together, our approach proposes that the pathogenesis of omphalocele may be better understood through a vascular framework centered on a potential arterial “switch” occurring during the physiological herniation and return of the midgut around the sixth gestational week. Such a rearrangement of the early arterial network could create regions of relative hypoperfusion and hypoxia, impairing the maturation and fusion of the abdominal wall. By integrating vascular (*CD31*, *CD34*, *ICAM-2*), muscular (*DUX4*), and extracellular matrix markers (Collagen I) and applying them to anatomically standardized sampling sites at the defect margin, our study transforms this hypothesis into a testable morpho-molecular model. Patterns of vessel density, angiogenic activation, muscle maturation, and collagen remodeling allow us to differentiate between a regional vascular mechanism, a primary mesodermal defect, or intrinsic muscular/ECM dysgenesis. Ultimately, this panel enables us to determine whether the abdominal wall is malformed because it was intrinsically programmed that way, or because it developed under compromised vascular conditions at a critical embryological moment—bringing us closer to clarifying the true etiopathogenesis of omphalocele.

## Figures and Tables

**Table 1:** Immunohistochemical score—supraumbilical samples (Cases 1–5).

Case	*ICAM-2*	*CD31*	*CD34*	Collagen I	*DUX4*
1	2	3	3	3	2
2	2	2	2	3	3
3	2	2	2	3	2
4	2	2	2	2	3
5	3	1	1	3	2

**Table 2:** Immunohistochemical score—subumbilical samples (Cases 6–11).

Case	*ICAM-2*	*CD31*	*CD34*	Collagen I	*DUX4*
6	2	2	3	3	3
7	2	2	2	2	3
8	2	2	3	2	3
9	2	1	3	3	3
10	2	3	3	2	2
11	2	3	3	3	3

**Table 3:** SEM Scoring by Case.

Case	SEM Score	Category (Estimated)	Morphologic/Functional Comment
1	3	(mild)	Discrete modifications; relatively preserved structure.
2	3	(mild)	Similarly to case 1, uniform appearance across fields
3	3	(mild)	Minimal alterations; no extensive degradation areas.
4	3	(mild)	Low-grade repetitive pattern.
5	3	(mild)	Stable, without major heterogeneity.
6	3	(mild)	Practically identical to cases 1–5.
7	5	(severe)	Evident alterations; more disorganized/porous architecture.
8	6	(severe)	The highest severity, marked heterogeneity, and degradation
9	5	(severe)	Severe, close to case 7.
10	4	moderate	Transition between mild and severe; clear lesions/defects, but not maximal.
11	4	moderate	Similarly to case 10.

**Table 4:** Descriptive Statistics for SEM.

Parameter	Value
*N* cases	11
Mean	3.82
Standard deviation (SD)	1.08
Median	3
Minimum- Maximum	3–6
Range	3

**Table 5:** Subgroup Statistics- Cases 1–6 vs. 7–11.

Subgroup	Cases	Mean	SD	Observation
Group A	1–6	3.00	0.00	Completely homogeneous group, all mild.
Group B	7–11	4.80	0.84	Heterogeneous group, predominantly moderate-severe.

**Table 6:** IHC and SEM scores in the supraumbilical region.

Case	*ICAM-2*	*CD31*	*CD34*	Collagen I	*DUX4*	SEM
1	2	3	3	3	2	3
2	2	2	2	3	3	3
3	2	2	2	3	2	3
4	2	2	2	2	3	3
5	3	1	1	3	2	3

**Table 7:** IHC and SEM scores in the subumbilical region.

Case	*ICAM-2*	*CD31*	*CD34*	Collagen I	*DUX4*	SEM-B
6	2	2	3	3	3	3
7	2	2	2	2	3	5
8	2	2	3	2	3	6
9	2	1	3	3	3	5
10	2	3	3	2	2	4
11	2	3	3	3	3	4

## Data Availability

Data are contained within the article.
